# MRAM: A Versatile Non-Volatile Memory for Next-Generation Computing

**DOI:** 10.3390/nano16130816

**Published:** 2026-07-01

**Authors:** Zhihan Wang, Haiwen Li, Sheng Jiang

**Affiliations:** School of Microelectronics, South China University of Technology, Guangzhou 510641, China

**Keywords:** MRAM, MRAM applications, future development trends

## Abstract

Magnetoresistive random-access memory (MRAM), as a promising non-volatile memory technology, has attracted extensive research interest owing to its unique combination of high operating speed, exceptional endurance, low standby power consumption, and CMOS process compatibility. In this review, we provide a comprehensive overview of the technological evolution of MRAM, spanning from Toggle-MRAM to spin-transfer torque (STT)-MRAM and then to spin–orbit torque (SOT)-MRAM. The working mechanisms, performance trade-offs, and integration potential of each generation are systematically summarized. Furthermore, the diverse applications of MRAM—including embedded systems-on-chip (SoCs), edge computing, aerospace and automotive electronics, artificial intelligence accelerators, neuromorphic computing, and hardware-level security—are thoroughly discussed. We also identify key challenges hindering large-scale commercialization, such as the trade-off between write energy and speed, process complexity, storage density constraints, and cost competitiveness. Finally, emerging research directions are proposed, emphasizing short-term priorities such as write current reduction and yield improvement, as well as long-term development strategies focusing on material–device–algorithm co-optimization and ecosystem establishment.

## 1. Introduction

The performance of modern computing systems is increasingly constrained by the fundamental limitations of conventional memory technologies, giving rise to the long-standing “memory wall” and “power wall” challenges [1,2]. Although mainstream memory technologies, such as dynamic random-access memory (DRAM), static random-access memory (SRAM), and non-volatile NAND Flash, each serve specific roles within the memory hierarchy, they all exhibit structural deficiencies that impede further scalability and efficiency [3,4]. DRAM, the dominant choice for main memory due to its high density and fast access characteristics, stores information as capacitive charge, which inevitably leaks over time. Consequently, frequent refresh operations are required to ensure data integrity, resulting in additional power consumption, increased latency, and reduced process scalability [5,6]. Its volatile nature, where data is immediately lost upon power interruption, further limits its applicability in emerging persistent memory scenarios. SRAM, commonly deployed as a cache, provides superior speed but incurs substantial area overhead owing to its six-transistor (6T) cell structure. As technology nodes scale, static leakage becomes increasingly pronounced, creating a significant bottleneck for energy-efficient design in large multi-core processors [7]. In contrast, non-volatile NAND Flash offers long-term data retention and is widely used in high-capacity storage. However, its intrinsic program/erase mechanism leads to highly asymmetric read/write performance, mandatory erase-before-write operations, limited endurance on the order of only tens of thousands of cycles, and the requirement for high programming voltages [4]. These factors collectively result in high power consumption and constrained durability. Together, these limitations hinder conventional memory technologies from simultaneously achieving the combined requirements of high speed, low power consumption, and high endurance demanded by next-generation computing architectures.

To address the shortcomings of existing memory technologies, both academia and industry have been actively exploring novel memory solutions that offer a balance of high speed, high density, non-volatility, low power, and high endurance. Magnetoresistive random-access memory (MRAM) [8] has emerged as a promising candidate. MRAM stores information using the spin property of electrons rather than charge. Its fundamental device structure is the magnetic tunnel junction (MTJ) [9], which encodes data via resistance states. This mechanism inherently provides non-volatility and eliminates the need for refresh operations, substantially reducing energy consumption. MRAM technology has followed a clear evolutionary path. Early Toggle-MRAM relied on magnetic fields to switch the magnetization of the free layer, facing scalability issues. The second-generation STT-MRAM directly manipulates magnetization using spin-polarized current, significantly reducing cell size and write energy. This enables embedded applications, such as replacement of eFlash, as well as initial commercialization of standalone memory. Current research has entered a third phase, exploring novel mechanisms for improved efficiency and reliability. These include SOT-MRAM, which features separate read and write paths, and Voltage-Controlled Magnetic Anisotropy MRAM (VCMA-MRAM), which modulates magnetic anisotropy via electric fields [10]. These technologies hold promise for further advancing write speed and power efficiency, offering potential hardware support for unified memory architectures and near-memory or in-memory computing.

**Figure 1:**
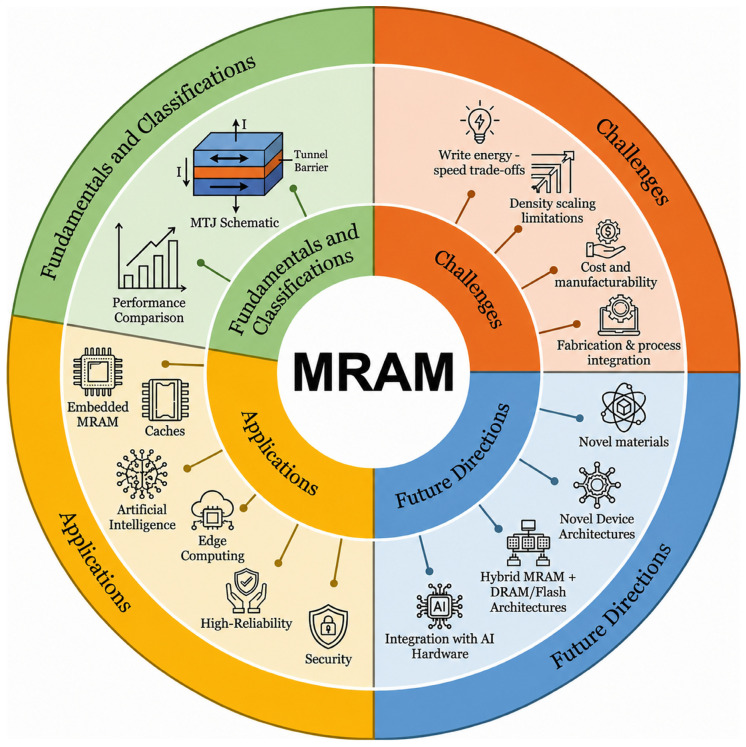
Organizational chart illustrating the research community landscape of MRAM, providing an overview of its structure and organization within this review.

This review aims to systematically trace the developmental trajectory of MRAM technology, provide an in-depth analysis of its physical mechanisms, and comprehensively assess its current research status and future potential across various application scenarios. As shown in Figure 1, we will first elucidate the fundamental principles and classifications of MRAM. We will then delve into specific applications in areas such as embedded systems, standalone memory, and cutting-edge explorations. Finally, we will summarize the key scientific and technological challenges that remain in MRAM development, while also outlining its future evolution path and potential for disruptive applications.

## 2. Fundamentals and Evolution of MRAM

### 2.1. Fundamentals of MRAM

**Figure 2:**
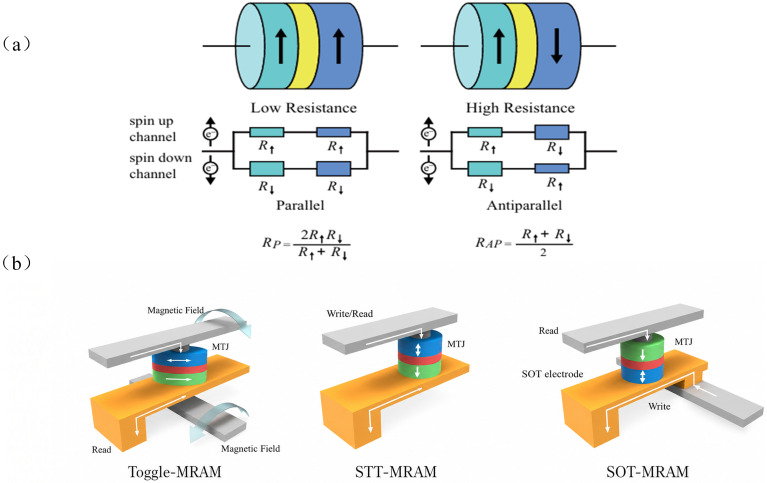
MRAM working mechanisms based on MTJ structures. (**a**) Two-channel model of an MTJ, illustrating spin-dependent transport in the parallel (low-resistance) and antiparallel (high-resistance) states, where the total resistance is determined by spin-up and spin-down conduction channels. Reprinted from Ref. [18]. (**b**) Schematic illustrations of three generations of MRAM technologies: Toggle-MRAM, which uses external magnetic fields for switching; STT-MRAM, which employs spin-transfer torque via a vertical current through the MTJ; and SOT-MRAM, which utilizes spin–orbit torque with separate write and read paths for improved performance. Reprinted from Ref. [19].

The MTJ comprises a reference layer with fixed magnetization, an insulating tunnel barrier (typically MgO), and a free layer whose magnetization direction can be switched. Data storage in the MTJ is based on the tunnel magnetoresistance (*TMR*) effect. When the magnetization directions of the reference and free layers are parallel, the electron spins are aligned; this increases the tunneling probability and results in a low-resistance state, denoted as $R_{P}$ in Figure 2a. Conversely, when the magnetizations are antiparallel, the electron spins are misaligned, reducing the tunneling probability and leading to a high-resistance state, $R_{AP}$. This measurable difference in resistance forms the physical mechanism for storing binary data (“0” and “1”) in MRAM. The *TMR*, defined by the equation below, is commonly used to quantify this resistance contrast:

(1) $$TMR=\frac{R_{AP}-R_{P}}{R_{P}}\times 100\%$$

For MRAM applications, the device merit of an MTJ depends not only on the existence of distinguishable resistance states, but also on two key parameters, namely *TMR* and the resistance–area product (RA). Early MTJs with amorphous AlOx barriers usually showed room-temperature *TMR* values below about 70%, resulting in a limited sensing margin and insufficient read robustness. The introduction of crystalline MgO barriers markedly improved the *TMR*, raising it to about 180% in epitaxial Fe/MgO/Fe junctions [11] and to about 220% in sputtered MgO-based MTJs [12]. Further optimization of CoFeB/MgO/CoFeB stacks, interface quality, and annealing conditions pushed the room-temperature *TMR* to 604% [13]. The continuous increase in *TMR* enlarged the resistance window between $R_{P}$ and $R_{AP}$, thereby improving the sensing margin, read reliability, and error tolerance. RA is defined as the product of the junction resistance and its effective area, usually expressed in $\Omega \cdot \mu m^{2}$. This parameter determines the effective impedance of the MTJ and directly affects read current, sensing speed, output voltage, and compatibility with CMOS access transistors. Device optimization in MRAM therefore requires a balance between *TMR* and RA rather than the maximization of either parameter alone. Low-resistance CoFeB/MgO/CoFeB MTJs have achieved a *TMR* of 138% with an RA of 2.4 $\Omega \cdot \mu m^{2}$ [14]. Perpendicularly magnetized MgO-based MTJs have maintained high magnetoresistance at an RA of about 4.4 $\Omega \cdot \mu m^{2}$ [15]. W-engineered perpendicular MTJs have reported a *TMR* of 249% with an RA of 7.0 $\Omega \cdot \mu m^{2}$ [16]. For low-RA perpendicular MTJs, the bias dependence of *TMR* must also be considered, since it further influences the output voltage and effective sensing margin during practical read operation [17]. Overall, the synergistic optimization of high *TMR* and an appropriate RA constitutes a fundamental device-level prerequisite for enabling high-speed, low-power, and high-reliability MRAM operations.

### 2.2. Evolution of MRAM

MRAM has been developed for more than three decades. Its device structure and working principle have undergone continuous innovation. Figure 2b shows the structural diagrams of three generations of MRAM. Toggle-MRAM, the first generation of MRAM, utilizes magnetic-field-induced switching via orthogonal word-line currents, with a synthetic antiferromagnet (SAF) free layer enabling deterministic Savtchenko toggle switching and effectively suppressing half-select disturbances. From the materials perspective, Toggle-MRAM is typically based on an MTJ stack that includes a pinned magnetic layer, an insulating tunnel barrier, and a SAF-based free layer. AlO_x_ tunnel barriers were commonly used in early field-switched MRAM, whereas MgO-based barriers later became important in the broader development of high-*TMR* MTJ technologies [20]. The SAF free layer generally consists of two ferromagnetic layers antiferromagnetically coupled through a thin non-magnetic spacer, such as Ru [21]. This coupled configuration is essential for Savtchenko switching, because the magnetizations of the two free-layer components can rotate in a controlled manner under the combined magnetic fields generated by the word and bit lines [22]. Because the write process is driven by magnetic fields rather than by charge trapping or repeated tunneling-current stress, this architecture offers ultra-high endurance and long-term data retention, making it suitable for radiation-hardened and mission-critical applications. Spurred by the promise of universal memory, intensive development of MRAM started in the mid-1990s [23]. Over the past decades, several research groups and industrial laboratories have contributed to the development of this technology [24,25,26,27]. As early as 2003, IBM introduced one of the first MRAM prototypes, characterized by high speed and endurance. In 2006, Freescale began mass production of 4 Mb Toggle-MRAM [28]. However, due to its magnetic-field switching architecture, this technology faced severe scalability limits, with commercial densities remaining much lower than those of mainstream semiconductor memories. It has therefore been mainly adopted by companies such as Honeywell and Cobham/CAES for aerospace and defense applications. The need for large field-generating write currents leads to high power consumption, increased circuit complexity, and poor scalability, ultimately driving the transition to STT-MRAM with superior energy efficiency and density.

Second-generation MRAM leverages the STT effect, in which a spin-polarized current flowing perpendicular to the MTJ plane transfers angular momentum to the free layer, enabling magnetization switching without external magnetic fields and eliminating field-generating write lines. This transition substantially improves cell scalability, energy efficiency, and CMOS integration. The material development of STT-MRAM is closely associated with perpendicular CoFeB/MgO/CoFeB-based MTJs. In a typical perpendicular STT-MRAM stack, the MgO tunnel barrier supports coherent spin-dependent tunneling and thereby provides a high tunnel magnetoresistance ratio [29]. The CoFeB/MgO interface also contributes interfacial perpendicular magnetic anisotropy, which is critical for maintaining thermal stability in scaled perpendicular MTJs [30,31]. The reference layer is often pinned by exchange bias or stabilized using a synthetic antiferromagnetic structure, whereas the free layer stores binary information through its parallel or antiparallel alignment with the reference layer. The physical basis of STT switching is the transfer of spin angular momentum from a spin-polarized current to the free-layer magnetization. During writing, a vertical current passes through the MTJ and becomes spin-polarized by the reference layer [32]. When the current exceeds the critical switching threshold, the spin-transfer torque overcomes magnetic damping and drives the free layer from one stable magnetic state to the other. Therefore, the CoFeB/MgO interface is not only responsible for readout through tunnel magnetoresistance but also strongly affects thermal stability, switching current, and write-error behavior in scaled STT-MRAM devices [29,30,31]. STT-MRAM achieves nanosecond-scale write speed, higher density, and endurance of 10^10^–10^12^ cycles, enabling its use as a replacement candidate for embedded Flash and expansion into standalone and persistent memory applications while retaining the standard 1T–1MTJ architecture. A major milestone in the commercialization of MRAM occurred in 2016, when Everspin launched the industry’s first 256 Mb STT-MRAM [33]. In 2019, Samsung began mass production of a 1 Gb embedded STT-MRAM based on a 28 nm FDSOI platform [34]. In 2022, Samsung advanced MRAM to the 14 nm FinFET node [35], while Everspin released 8–64 Mbit SPI-interface MRAM products for industrial Internet of Things (IoT) and embedded applications [36]. As manufacturing processes mature and capacity scales up, MRAM has expanded beyond niche aerospace applications into mainstream markets such as IoT, artificial intelligence, automotive electronics, and persistent memory. Nevertheless, the shared read/write current path of STT-MRAM introduces read-disturb concerns and write-error-rate (WER) challenges. These issues become more pronounced at scaled technology nodes, where reduced thermal stability, MgO barrier reliability, and the trade-off among current density, latency, and retention must be carefully balanced.

SOT-MRAM, regarded as the third generation of MRAM, adopts a three-terminal architecture that decouples read and write paths and enables magnetization switching via SOT generated by in-plane currents in a heavy-metal layer, thereby eliminating read disturbance. In SOT-MRAM, the MTJ is integrated with an additional spin-source layer adjacent to the free layer [37,38]. This spin-source layer is commonly formed using materials with strong spin–orbit coupling, such as Pt, Ta, W, TaW alloy or β-W [39,40,41], and its material properties directly determine the charge-to-spin conversion efficiency, write current density, and Joule heating [38,42]. During writing, an in-plane charge current flows through the spin-source layer and is converted into a transverse spin current through the spin Hall effect or interfacial spin–orbit coupling [37,38]. The resulting spin accumulation at the interface exerts damping-like and field-like torques on the adjacent free layer [40]. These torques enable magnetization reversal without forcing the write current through the MgO tunnel barrier [37,38,39,40,41,42,43,44]. This separation between the lateral write path and the vertical read path is the central device-level advantage of SOT-MRAM, because it suppresses read disturbance and reduces electrical stress on the tunnel barrier [37,38]. However, deterministic field-free switching usually requires symmetry breaking through material, structural, or magnetic design [37,38,39,40,41,42]. In addition, the resistivity of the spin-source layer, interface transparency, spin Hall angle, and electromigration reliability remain critical factors for array-level integration [38,42]. Depending on the orientation of the magnetic easy axis relative to the write current, SOT-MRAM switching can be classified into Type Z (out-of-plane easy axis), Type Y (in-plane transverse easy axis), and Type X (easy axis parallel to the current, typically requiring an assist field), each exhibiting distinct switching dynamics [43]. Among these configurations, Type-Z SOT-MRAM is particularly attractive for high-density memory because its perpendicular magnetic anisotropy is compatible with scaled p-MTJs and provides favorable thermal stability. Its main challenge is deterministic field-free switching, since conventional heavy-metal layers mainly generate in-plane spin polarization. Current approaches therefore employ tilted anisotropy, exchange bias, compositional gradients, or low-symmetry spin-source materials to introduce built-in symmetry breaking or an out-of-plane spin-polarization component [45,46]. Recent van der Waals heterostructures have also demonstrated room-temperature field-free switching through unconventional out-of-plane torque [47]. Further development still requires improvements in wafer-scale growth, thermal robustness, process uniformity, and CMOS-compatible integration [48]. Although SOT-MRAM demonstrates sub-nanosecond switching speed and ultra-high endurance, it remains largely in the research and early development stage due to challenges including high write current density, increased cell footprint, and integration complexity. Since 2018, third-generation SOT-MRAM has gained attention for its separate read/write path and ultrafast switching. Related work from Beihang University and its collaborators has played an important role in advancing SOT-MRAM from device-level studies toward manufacturable technologies: field-free switching of a perpendicular magnetic tunnel junction through the interplay of spin–orbit torque and spin-transfer torque was experimentally demonstrated in 2018 [49], high-performance SOT-MTJ devices were integrated on a 200 mm wafer manufacturing platform in 2022 [50], and a manufacturable 1 Kb SOT-MRAM multiplexer array was further reported in 2023 [51]. In 2025, a collaboration between NYCU, TSMC, and ITRI demonstrated a 64 kb β-phase tungsten SOT-MRAM array achieving ~1 ns switching, over 10-year retention, and thermal stability up to 700 °C—marking a critical step toward practical applications [52]. We have compiled a timeline of MRAM product production and technology development from 2005 to the present in Table 1.

### 2.3. Performance Comparison

From a technological evolution standpoint, Toggle-MRAM, STT-MRAM, and SOT-MRAM represent successive milestones in the advancement of MRAM technology, each introducing breakthroughs in write mechanisms, power efficiency, integration density, and reliability. Table 2 provides a comparative summary of key metrics across major memory technologies, highlighting the performance, scalability, and integration advantages of STT-MRAM and SOT-MRAM relative to Toggle-MRAM, Flash, DRAM, and SRAM.

In this review, the evolution of MRAM reflects a clear transition from field-driven to spin-transfer-torque-driven to spin–orbit-torque-driven architectures. Toggle-MRAM establishes the industrial foundation with its superior reliability; STT-MRAM achieves a balanced trade-off between density and energy efficiency, enabling large-scale commercialization; and SOT-MRAM approaches the theoretical limits of speed and endurance, positioning itself as a key enabler for next-generation unified memory and computing systems. Future research will likely focus on lowering SOT write energy, achieving higher *TMR* values, and improving thermal stability, ultimately driving MRAM toward greater capacity, lower power consumption, and broader applicability in emerging computing paradigms.

### 2.4. Fabrication Process of SOT-MRAM

**Figure 3:**
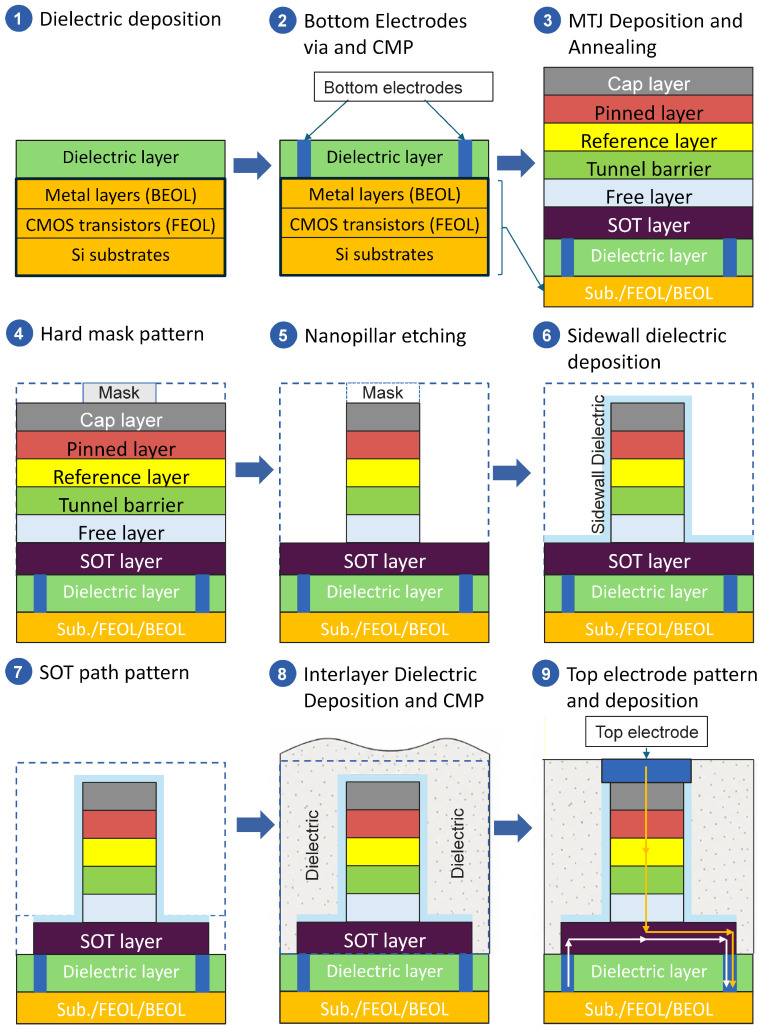
Fabrication process of SOT-MRAM.

The fabrication of SOT-MRAM involves the hybrid integration of MTJ and CMOS processes. The MTJ fabrication processes can be carried out after the multilevel metal interconnects in the CMOS back-end-of-line (BEOL) are formed. This section focuses on the representative MTJ back-end-of-line process (which may vary across different manufacturers and foundries). Figure 3 shows the typical fabrication process of SOT-MRAM. The key steps are detailed as follows:

① **Dielectric deposition:** After the conventional CMOS process is completed, a dielectric material (SiO_2_, SiN_x_) with an appropriate density and thickness is deposited on the Si substrate, where the CMOS transistors (including front-end-of-line (FEOL) devices) and BEOL metal interconnects have already been fabricated, thereby forming a dielectric layer to support the subsequent MTJ devices.
② **Bottom electrode via and CMP:** Bottom electrode via structures are formed by etching, enabling electrical connection between the bottom electrode and the underlying BEOL metal layer; metal is deposited to fill the vias and form the bottom electrode; then, chemical mechanical polishing (CMP) is performed to reduce the surface roughness (ideally below 0.2 nm), providing a flat and high-quality surface for the growth of the SOT-MTJ device, which is essential for ensuring the performance of SOT-MRAM.
③ **MTJ deposition and annealing:** Since the SOT layer serves as the path for the write current to generate spin current that drives the magnetization switching of the free layer, SOT-MRAM adopts a top-pinned configuration, in which the pinned layer and reference layer are located near the top electrode, while the free layer is positioned adjacent to the SOT layer. The basic functional layer structure is illustrated in Figure 3③. A multilayer stack including the SOT layer, MTJ layers, and capping layer is deposited by magnetron sputtering. After stack deposition, vacuum annealing is usually performed under a magnetic field for better *TMR* values. During this step, current-in-plane tunneling (CIPT) measurement is required to monitor key properties of the film stack, such as the *TMR* ratio and RA.
④ **Hard mask pattern:** A dielectric layer, normally SiO_2_, SiNx, or SiC, is deposited to serve as the hard mask later on. Then, photolithography is performed to define the hard mask pattern, then the hard mask layer is etched, followed by complete removal of the photoresist.
⑤ **MTJ nanopillar etching:** The MTJ multilayer stack is etched and the hard mask is partially (the remaining part will be removed later on) or fully consumed during the etching. The etching process must be precisely stopped above the SOT channel layer, while ensuring that the free layer is completely etched.
⑥ **Sidewall dielectric deposition:** An in situ sidewall dielectric layer is deposited to prevent moisture, oxygen, and other contaminants from degrading the MTJ layers.
⑦ **SOT layer pattern:** Photolithography is performed to define the SOT channel pattern, and then to etch the SOT layer.
⑧ **Interlayer dielectric deposition and CMP:** A dielectric layer is deposited to fill the gaps and isolate the MTJs, then CMP is first performed to planarize the surface.
⑨ **Top electrode pattern and deposition:** An interlayer dielectric layer is deposited, photolithography is performed to define the top electrode via pattern above the MTJ; the dielectric layer is etched to form the top electrode via structure; then the top-electrode metal is deposited. Finally, the SOT-MRAM is ready, and the write current path is indicated by white arrows, whereas the read path is indicated by yellow arrows. Depending on the interconnection requirements, more than one metal layer may be formed after the MTJ. In addition, to meet the functional requirements of chip packaging, wire-bonding pads and a passivation layer need to be formed at the topmost level.

## 3. Applications of MRAM

**Figure 4:**
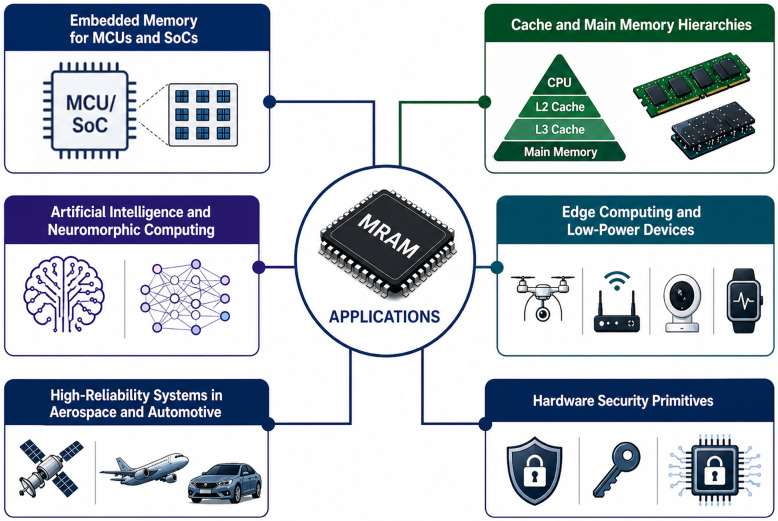
Applications of MRAM.

The unique combination of non-volatility, high speed, and low power consumption positions MRAM as a versatile technology for a wide range of application scenarios. As shown in Figure 4, we discuss its applications across six representative domains: (1) embedded memory for microcontrollers and SoCs, (2) cache and main memory hierarchies, (3) artificial intelligence and neuromorphic computing platforms, (4) edge computing and low-power devices, (5) high-reliability systems in aerospace and automotive environments, and (6) hardware security.

### 3.1. Embedded MRAM

In modern SoC design, embedded memory is a critical subsystem that directly affects overall power efficiency, computational performance, and system reliability. With SoC integration levels rapidly increasing and workloads becoming more heterogeneous, embedded memory is expected to deliver not only high-speed access and low power consumption, but also improved non-volatility, area efficiency, and CMOS process compatibility.

Traditionally, SRAM and eFlash have been the two dominant embedded memory solutions. SRAM, featuring nanosecond-level access latency and full CMOS compatibility, is widely adopted in caches and register. However, its 6T structure results in a large cell area and high static power leakage, while the lack of non-volatility prevents data retention upon power loss. eFlash, in contrast, provides power-off data retention, but suffers from slow write operations and high programming voltages. Additionally, it requires 6–7 additional mask layers for fabrication and a dedicated high-voltage module, leading to increased fabrication cost and process complexity and poor compatibility with advanced logic nodes [85,86]. In response to these limitations, STT-MRAM has emerged as a next-generation embedded non-volatile memory (eNVM), combining nanosecond-level access speed, non-volatility, negligible static leakage, and high endurance [85]. MTJs, the key memory cell of MRAM, can be monolithically integrated into the back-end-of-line (BEOL) with only three additional masks, significantly simplifying fabrication compared to eFlash [86,87].

The industrial-grade reliability of embedded STT-MRAM has been validated through silicon demonstrations. In 28 nm FDSOI logic processes, embedded STT-MRAM macros operate over a temperature range from −40 °C to 125 °C, tolerate 260 °C solder reflow, and maintain stable write operation under external magnetic field disturbances up to 550 Oe [87]. Through MTJ stack and patterning optimizations, tunnel magnetoresistance ratios exceeding 200% are achieved, enabling read margins beyond 25σ in 8 Mb macro arrays. Combined reliability assessments verify endurance exceeding 10^6^ write cycles, 1000 h high-temperature retention, and 10-year data retention, while error-correcting codes and redundancy are employed to ensure high yield at the array and system levels [88].

**Figure 5:**
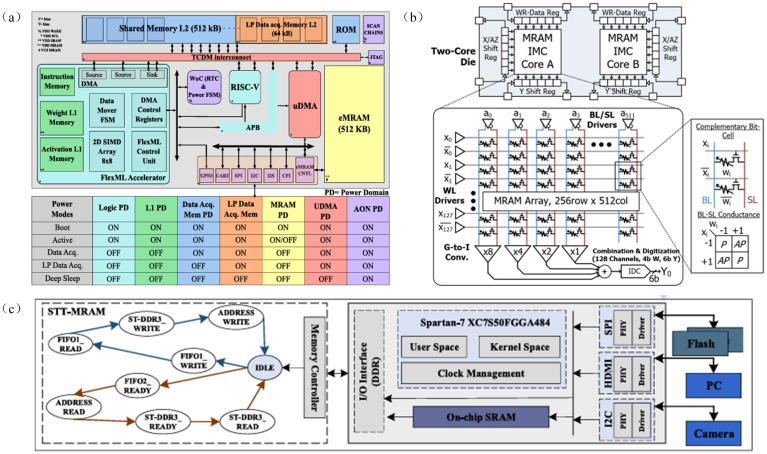
Embedded MRAM. (**a**) TinyVers, is a heterogeneous SoC consisting of a single-core RISC-V processor, a Flexible Machine Learning (FlexML) accelerator, a 512 kB shared level−2 (L2) SRAM memory, a micro−DMA (uDMA) for data movement between peripherals/memory, a 512 kB eMRAM for non−volatile storage, and a Wake−up Controller (WuC) for power management. Reprinted from Ref. [90]. (**b**) A block diagram of the presented MRAM IMC prototype, which includes two IMC macros implemented on one die, in order to evaluate noise interference within a parallelized macro and across parallel macros. Reprinted from Ref. [91]. (**c**) The architecture of the proposed nonvolatile instant−on/off system based on STT-MRAM, which can instantly power on/off under different conditions of the harvested energy. Reprinted from Ref. [92].

Subsequent technology generations extend these reliability results to higher densities and a wider operating envelope. Shimoi et al. demonstrated a 22 nm, 32 Mb embedded STT-MRAM macro for high-end microcontrollers, featuring 5.9 ns random read, 7.4 MB/s write throughput, and reliable operation up to 150 °C enabled by a boosted cross-coupled sense amplifier and a fast-write scheme [89]. At the system level, in Figure 5a, Jain et al. integrated state-retentive embedded MRAM into the TinyVers heterogeneous machine-learning SoCs as non-volatile storage for boot code and model parameters, enabling aggressive duty cycling with deep-sleep power consumption reduced to 1.7 µW [90].

Beyond conventional storage applications, embedded MRAMs fabricated in advanced technology nodes are increasingly repurposed as compute-capable arrays. At the array operation level, write margin robustness is evaluated through write voltage–access time operating windows. Myung S et al. presented shmoo plots under different operating conditions, showing wide and well-defined pass regions that indicate a sufficient write margin across process, voltage, and temperature variations [91]. Later on, Deaville, P et al. demonstrated a 14 nm MRAM-based multi-bit analog in-memory computing macro in which stacked MTJs implement multi-bit weights, enabling calibrated bit-parallel multiply–accumulate operations with performance reaching 18.29 TOPS/mm^2^ and energy efficiency of 340.8 TOPS/W in Figure 5b [92]. Deaville et al. further reported a fully row- and column-parallel in-memory computing macro implemented in a foundry 22 nm MRAM, employing differential readout and conductance-to-current conversion to enhance signal-to-noise ratio and robustness against device variability [93]. Beyond reliability-oriented memory operation, recent demonstrations show that MTJ critical dimensions can be scaled below 50 nm while maintaining sub-ppm bit-error rates. Such scaling enables both RAM-like operation with write endurance exceeding 10^14^ cycles and Microcontroller Unit (MCU)-oriented operation with endurance beyond 10^6^ cycles and data retention exceeding 20 years at temperatures above 150 °C [94]. Recent automotive-grade eMRAM demonstrations further push endurance and energy-efficiency limits, reporting write energies down to 10 pJ/bit and endurance beyond 10^12^–10^14^ cycles while supporting reliable operation up to 150 °C [95].

STT-MRAM is also particularly well suited for IoT edge devices and mobile SoCs. As shown in Figure 5c, an STT-MRAM-based instant-on/off system has been demonstrated in low-power IoT nodes, enabling devices to tolerate intermittent power without state loss [96]. In mobile SoCs, STT-MRAM can function as non-volatile caches, tightly coupled memory (TCM), or replacement for fast ROM blocks, reducing static power consumption while preserving performance. Moreover, STT-MRAM-enabled AI/ML accelerators in mobile/edge designs achieve reduced leakage, area savings, and enhanced robustness under process and temperature variation [97].

These use cases further strengthen the argument that STT-MRAM is not just a candidate for embedded control systems, but also a transformative memory solution for low-power IoT and mobile platforms. Embedded STT-MRAM successfully bridges the gap between SRAM’s speed and eFlash’s non-volatility, providing an energy-efficient, scalable, and reliable solution for next-generation SoCs. Its industrial validation and process maturity indicate readiness for deployment in diverse domains, including automotive electronics, industrial IoT, and smart embedded controllers.

### 3.2. MRAM in Caches and Main Memory

**Figure 6:**
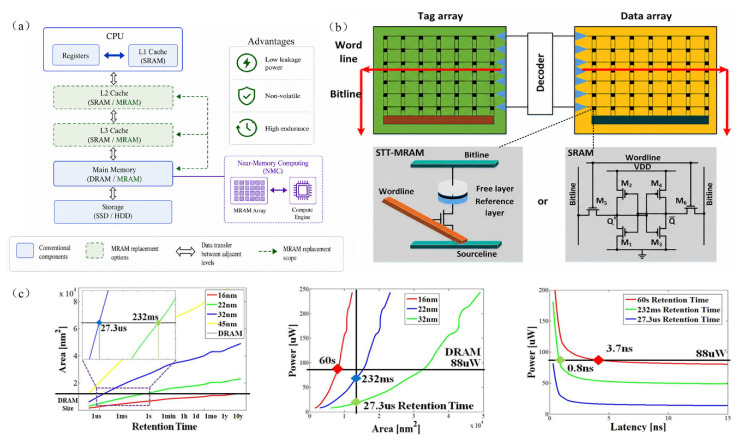
MRAM in caches and main memory. (**a**) Basic schematic structure of the computer system, including CPU, cache, main memory and storage. MRAM can replace conventional memory in L2/L3 caches and main memory, while enabling near-memory computing for improved energy efficiency. (**b**) Cache architecture with separated tag and data arrays, MRAM can replace conventional SRAM as the memory cell in the data array. Reprinted from Ref. [98]. (**c**) Comparison of STT-MRAM and DRAM under different retention times, showing trade-offs in area, power, and latency. Reprinted from Ref. [99].

As semiconductor technology scaling approaches its physical and energy limits, cache and main memory subsystems have become dominant determinants of computing system performance and energy efficiency. Traditional SRAM and DRAM technologies, despite their high speed, suffer from static leakage, scalability issues, and inherent volatility under deep submicron processes. Figure 6a illustrates the integration of MRAM within the memory hierarchy, where it serves as a flexible replacement for conventional SRAM in cache levels and a potential candidate for main memory, while enabling near-memory computing to reduce data movement overhead.

#### 3.2.1. L2/L3 Cache Substitution

At intermediate cache levels, STT-MRAM offers a compelling trade-off between energy efficiency, latency, and density. While SRAM provides nanosecond-level speed, its 6T structure induces high static power dissipation, particularly in multi-megabyte on-chip caches. In contrast, STT-MRAM nearly eliminates standby leakage and allows denser array configurations. Simulation-based studies by Zhang et al. indicated that for cache capacities exceeding 32 MB, STT-MRAM achieves an overall 42% reduction in energy consumption compared to SRAM while maintaining comparable access latency [98]. Figure 6b illustrates how STT-MRAM cells can replace conventional SRAM in cache implementations, highlighting their density advantage and the potential for hybrid cache architectures [98]. Experimental implementations further confirm this potential: the Samsung Embedded MRAM macro demonstrates adaptive bias control and ECC-assisted endurance optimization in a 28 nm process, achieving approximately 20% write energy reduction and 15–40% area savings compared with eFlash [85]. Additionally, ROM-embedded STT-MRAM (R-MRAM) structures have been proposed to integrate read-only tables and cache memory into a unified macro, achieving higher bit density without degrading cache-mode access latency—particularly beneficial for microcode or lookup-table applications in L2/L3 caches [88]. Despite these advantages, several critical challenges hinder the large-scale deployment of STT-MRAM as a direct SRAM replacement. In particular, the relatively high write current leads to increased write energy and latency variability, while reliability concerns—including WER, read disturbance, and retention degradation—become more pronounced at scaled technology nodes. Furthermore, the integration of MTJs into standard CMOS processes introduces additional fabrication complexity and cost overhead. These limitations necessitate careful architectural and circuit-level co-optimization rather than straightforward substitution.

The last-level cache (LLC), which in many modern processors corresponds to the L3 cache, represents the most extensively explored use case for MRAM-based storage. Marinelli et al. conducted a comprehensive microarchitectural exploration of STT-MRAM LLC configurations and demonstrated that through optimizing cache associativity, banking, and Miss Status Holding Register (MSHR) management, LLC energy consumption can be reduced by up to 60% with less than 8% performance degradation [100]. Complementary works further exploit perpendicular MTJ (pMTJ) stacks to improve area efficiency and energy scalability in non-volatile LLC implementations [101]. Hybrid cache architectures combining SRAM and STT-MRAM banks have also been widely investigated. By mapping read-dominant data to MRAM while retaining write-intensive blocks in SRAM, these schemes effectively mitigate write-energy and endurance limitations, achieving balanced throughput and reliability [102]. Cheshmikhani et al. proposed a system-level reliability modeling framework that captures the combined impact of multiple error mechanisms, enabling reliability-aware cache management and error-correcting code design [103].

#### 3.2.2. From Main Memory Integration to Near-Memory Storage

Beyond cache-level deployment, MRAM has also been explored as a candidate for non-volatile main memory and near-memory storage. Jin et al. demonstrated that, with circuit-level optimization, STT-MRAM-based main memory systems can reduce total power consumption by up to 66% compared with DRAM while achieving competitive access latency under specific operating conditions [99]. Figure 6c illustrates the optimization trends in area, power, and latency of STT-MRAM by relaxing the retention time, indicating that MRAM can complement or partially replace DRAM in energy-constrained systems.

In accelerator-centric platforms, cross-layer optimization frameworks such as DeepNVM++ have explored the use of STT-MRAM and SOT-MRAM in deep neural network memory hierarchies. Compared with SRAM- and DRAM-based designs, this approach can improve the energy-delay product (EDP) and area efficiency [104]. Similarly, Han et al. proposed an advanced hybrid MRAM-based GPU cache system for graphics processing, achieving up to 28% speed improvement, 56% energy reduction, and 66.45% leakage power optimization, highlighting MRAM’s applicability to high-performance computing workloads [102].

At this stage, MRAM primarily serves as a near-memory storage medium rather than an active computing element. However, its proximity to processing units and intrinsic non-volatility naturally motivate the transition toward more aggressive memory-centric computing paradigms.

#### 3.2.3. MRAM-Based Near-Memory Computing

Building upon MRAM’s successful deployment in cache hierarchies and near-memory storage, recent research has extended its role toward near-memory computing (NMC), in which selected computations are offloaded to memory-adjacent structures to reduce data movement. The energy benefit of NMC originates from the large difference between arithmetic and data-access costs. At the 45 nm technology node, a 32-bit integer addition consumes approximately 0.1 pJ, whereas a 64-bit access to an 8 kB cache requires about 10 pJ and a 64-bit DRAM access consumes approximately 1.3–2.6 nJ [83]. These values vary with the technology node and memory organization, but they show that repeated processor–memory transfers can consume substantially more energy than the arithmetic operation itself.

Unlike storage-centric use cases, MRAM-based NMC emphasizes application-level acceleration by exploiting data locality and non-volatility. Placing computation close to the MRAM array reduces external-memory accesses and the movement of operands and intermediate results. In a representative STT-MRAM-based NMC architecture for sparse matrix–vector multiplication, the optimized readout circuit achieved an energy consumption of 0.242 pJ/bit and a memory bandwidth of 26.7 GB/s. Depending on matrix sparsity, the proposed accelerator reported up to a 64× improvement in energy consumption and up to a 1120× reduction in execution latency compared with the unoptimized implementation [105]. These results are workload- and architecture-dependent, but they provide a quantitative example of the energy savings obtained by reducing data movement and irregular memory accesses.

Representative MRAM-NMC applications include security-aware processing, embedded and IoT systems, neural network inference, intermittent computing, and approximate computing. Near-memory shift-and-rotate operations and data-encrypted computation macros demonstrate high-bandwidth and energy-efficient support for cryptographic workloads [106,107]. In always-on and IoT platforms, MRAM enables state-retentive sleep modes and instant wake-up, significantly extending system lifetime [108]. For neural network inference and energy-harvesting systems, MRAM-NMC architectures reduce external memory access and enable computation recovery under intermittent power supply [109,110]. In addition, approximate computing techniques exploit intrinsic write-accuracy trade-offs in STT-MRAM to achieve further energy savings at the memory level [111,112,113].

These studies illustrate that MRAM has evolved from a device-level memory innovation into a system-level enabler spanning cache hierarchies, main memory, and near-memory computing applications, positioning it as a key building block for energy-efficient heterogeneous SoCs and AI accelerators.

### 3.3. MRAM in Artificial Intelligence

The application of MRAM in artificial intelligence has progressed from basic functional demonstrations to advanced system integration. Early proof of concept showed that STT-MRAM arrays could perform in situ logic and arithmetic through multi-row activation and current summation, enabling vector operations directly inside memory [114]. The initial results, obtained largely from small-scale test structures and architecture-level simulations, indicated that MRAM could serve functions beyond conventional storage, motivating further compute-in-memory (CIM) research. During the same period, MRAM was also explored as a synaptic device for neuromorphic computing, drawing on its non-volatility and CMOS compatibility.

**Figure 7:**
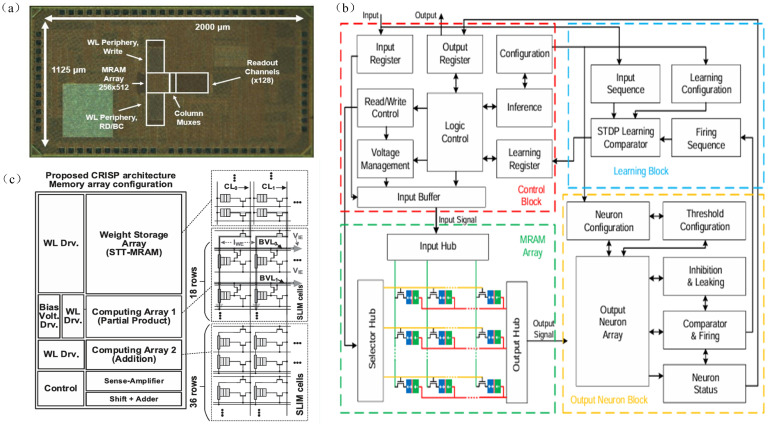
MRAM in artificial intelligence. (**a**) Optical micrograph of a fabricated STT-MRAM array with peripheral circuits, illustrating large-scale integration capability for memory and compute functions. Reprinted from Ref. [117]. (**b**) MRAM-based neuromorphic computing system architecture, including control block, STDP-based learning block, MRAM synaptic array, and output neuron circuits, supporting unsupervised learning and spike-based processing. Reprinted from Ref. [119]. (**c**) Proposed CRISP architecture based on SOT-MRAM, integrating weight storage and computing arrays to enable in-memory multiplication and accumulation without analog-to-digital conversion. Reprinted from Ref. [122].

Hardware demonstration and architectural diversification saw the first fabricated STT-MRAM CIM macros and a wealth of architecture-level proposals. Prior to silicon validation, simulation-based studies, such as Stoch In-Memory Computing (IMC) [115] and cross-layer analyses of STT-MRAM IMC, provided early performance projections and design insights that helped to guide subsequent hardware implementations [116]. As shown in Figure 7a, a 128 kb maximally row-parallel MRAM IMC macro was fabricated in a 22 nm FD-SOI process. The macro achieves 5.1 TOPS/W and 758 GOPS/mm^2^ while maintaining 90% CIFAR-10 inference accuracy, providing silicon-level evidence for large-scale MRAM-based AI accelerators [117]. In neuromorphic computing, fabricated MRAM synaptic arrays and neuron circuits have also been reported. For example, STT-MRAM devices were used to demonstrate spike-timing-dependent plasticity (STDP) [118], while stochastic MRAM switching enabled unsupervised STDP learning with 90% MNIST accuracy [119]. As shown in Figure 7b, the integrated MRAM-based neuromorphic architecture consists of a control block, an STDP-based learning block, an MRAM synaptic array, and output neuron circuits. Furthermore, MRAM synapses with multi-level weights achieved <3% MNIST error [120]. Concurrently, a number of CIM architectures were introduced at the design and simulation level. The NAND-SPIN architecture organized MRAM bit-cells into a NAND-like structure with page-wise activation for parallel CNN inference [121]. The SLIM and CRISP schemes employed SOT-MRAM to perform analog-to-digital conversion (ADC)-free matrix multiplication [122] as shown in Figure 7c, where the CRISP architecture integrates weight storage and computing arrays to enable in-memory multiplication and accumulation without ADC. A time-domain STT-MRAM CIM approach encoded logic and accumulation as pulse widths on shared bitlines [123]. The SIMPLY+ framework targeted improved read margin and lower bit-error rates under device variability [124]. In 2025, the Falcon event-driven object-tracking SoC, fabricated in 28 nm FDSOI technology, integrated heterogeneous MRAM computing blocks—processing-in-memory (PIM) for CNN encoders and NMC for attention-flow processing—and reached macro and system energy efficiencies of 750.18 TOPS/W and 16.34 TOPS/W [125].

These developments show that MRAM has evolved from a storage technology into a capable platform for energy-efficient AI computation. Through co-design across materials, circuits, and algorithms, researchers have demonstrated scalable and reliable MRAM-based neuromorphic systems, and ongoing work on device–circuit–algorithm synergies is expected to further expand the role of MRAM in brain-inspired computing.

### 3.4. MRAM for Edge Computing and Wearables

**Figure 8:**
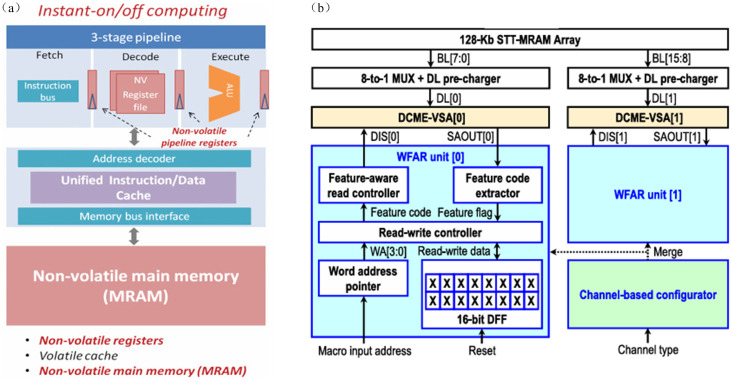
MRAM in edge computing. (**a**) Instant-on/off computing architecture based on STT-MRAM, where non-volatile registers and main memory enable retention of processor states across power interruptions, allowing rapid system recovery without full reboot. Reprinted from Ref. [126]. (**b**) STT-MRAM-based near-memory computing macro, integrating MRAM arrays with feature-aware read controllers and channel-based configurators to perform data access, feature extraction, and accumulation within the memory, enabling efficient edge-AI processing. Reprinted from Ref. [127].

Edge computing requires memory systems that combine ultra-low standby power, fast wake-up/restore capability, and non-volatility. MRAM naturally meets these requirements, offering zero standby leakage, high endurance, and instant data retention without power, making it highly suitable for energy-constrained IoT and wearable devices. In scenarios such as energy-harvesting or battery-limited nodes, frequent power interruptions necessitate rapid system recovery with minimal overhead. Non-volatile processor (NVP) architectures based on MRAM address this challenge by backing up processor states before power loss and restoring them upon wake-up, thereby significantly reducing energy consumption and latency associated with state recovery [126]. As illustrated in Figure 8a, MRAM is integrated into the processor pipeline as non-volatile registers and main memory, allowing instruction states, register contents, and intermediate execution data to be retained across power interruptions, thereby enabling instant system resumption. This establishes MRAM as a fundamental building block for reliable instant-on/off operation in edge computing systems.

Beyond system-level power management, MRAM also enables efficient data processing close to memory, which is critical for edge-AI workloads. An NMC architecture based on STT-MRAM demonstrates substantial improvements in data throughput and computational efficiency, achieving up to 64× energy efficiency gains and over three orders of magnitude latency reduction for sparse workloads [105]. Similarly, SOT-MRAM-based PIM architectures have been proposed to support compressed deep neural network (DNN) inference in resource-constrained environments, reducing data movement overhead while maintaining high computational efficiency [128]. These architectures highlight the role of MRAM in enabling low-power, high-efficiency AI processing directly within edge devices.

Recent hardware implementations further validate the practicality of MRAM in real-world edge platforms. The Vega SoC, designed for IoT end-nodes, integrates on-chip MRAM to support state-retentive sleep modes and near-sensor DNN acceleration, achieving ultra-low-power cognitive wake-up and fully on-chip inference [129]. In addition, an 8 Mb STT-MRAM NMC macro has demonstrated energy-efficient edge-AI inference using sparsity-aware techniques within a compact embedded system [127]. In Figure 8b, the STT-MRAM near-memory computing macro integrates MRAM arrays with feature-aware read controllers and channel-based configurators, enabling data access, feature extraction, and accumulation operations to be performed directly within the memory macro. At a higher level of integration, the Siracusa heterogeneous XR processor incorporates an MRAM-based neural engine tightly coupled with on-chip MRAM memory, achieving significant improvements in both throughput and energy efficiency, compared to off-chip memory solutions [130]. These system-level designs collectively demonstrate that MRAM can be effectively integrated into edge-AI platforms, enabling scalable and energy-efficient computation under strict power constraints. However, such gains are often workload-dependent, and many designs remain at the architecture or simulation level, with limited validation across diverse real-world edge scenarios.

Overall, the convergence of non-volatile system design, near-memory computing architectures, and MRAM-enabled edge processors indicates that MRAM is not only a memory technology but also a key enabler of efficient edge computing. Nevertheless, practical applications still require further optimization, particularly in reducing write energy and latency under high-frequency workloads, improving tolerance to device variability and reliability issues, and achieving cost-effective integration with CMOS technologies. Continued progress in device–circuit–architecture co-design is therefore essential to fully realize the potential of MRAM in next-generation intelligent edge systems.

### 3.5. MRAM in High-Reliability Domains

In aerospace, military, and automotive systems, memory components must survive harsh radiation, extreme temperatures, and vibrations and demand ultra-high endurance and data integrity. Conventional memories are vulnerable to single event upsets (SEUs), total ionizing dose (TID) degradation, and limited endurance. MRAM, by virtue of its magnetic storage mechanism, demonstrates intrinsic immunity to many radiation effects, virtually unlimited cyclic endurance, and robust performance under extreme conditions—making it a leading candidate for high-reliability non-volatile memory in such demanding applications.

One of the most compelling advantages of MRAM is its resilience to radiation. MTJs are inherently insensitive to charge-based disruptions, making them resistant to SEUs. Montoya et al. experimentally validated that nanoscale MTJs retain their switching and *TMR* characteristics after high-dose gamma and neutron irradiation, confirming their robustness in radiation environments [131]. Everspin’s commercial MRAM is independently tested to endure >1 Mrad without inducing hard errors and shows SEL tolerance to Linear Energy Transfer (LET) at approximately 84 MeV·cm^2^/mg. Their radiation-hardened offerings are positioned for aerospace and defense markets. Katti et al. described MRAM devices designed with radiation-hard circuits and high cycling endurance, tailored for space missions [132]. Honeywell also promotes radiation-hardened MRAM ICs for spacecraft use, citing their ability to resist ionizing environments for boot code, configuration memory, and data logging. Avalanche Technology’s “Space Grade” MRAM devices have passed NASA heavy-ion tests up to 1 Mrad without parameter degradation, Single Event Latch-up (SEL) LETth > 85.4, Single Event Functional Interrupt (SEFI) thresholds >120, and no permanent faults post-irradiation.

MRAM demonstrates a compelling combination of radiation resistance, near-unlimited endurance, retention across temperature extremes, and mechanical robustness, making it ideally suited for high-reliability domains such as aerospace, military, and automotive electronics. Though trade-offs remain (density, cost, and integration), MRAM’s unique physics give it a distinct advantage over charge-based memories in mission-critical, harsh-environment systems.

### 3.6. MRAM in Security Applications

With the growing demand for hardware-level security in IoT, edge AI, automotive, and defense-grade systems, MRAM has emerged as a compelling platform for implementing cryptographic primitives, particularly Physical Unclonable Functions (PUFs) and True Random Number Generators (TRNGs). Unlike charge-based memories, MRAM’s stochastic magnetization switching and thermal noise properties offer natural entropy sources suitable for generating secure keys and random numbers. Furthermore, MRAM’s non-volatility, process compatibility, and robustness against environmental fluctuations make it highly attractive for secure boot, identity authentication, key storage, and anti-tampering applications.

**Figure 9:**
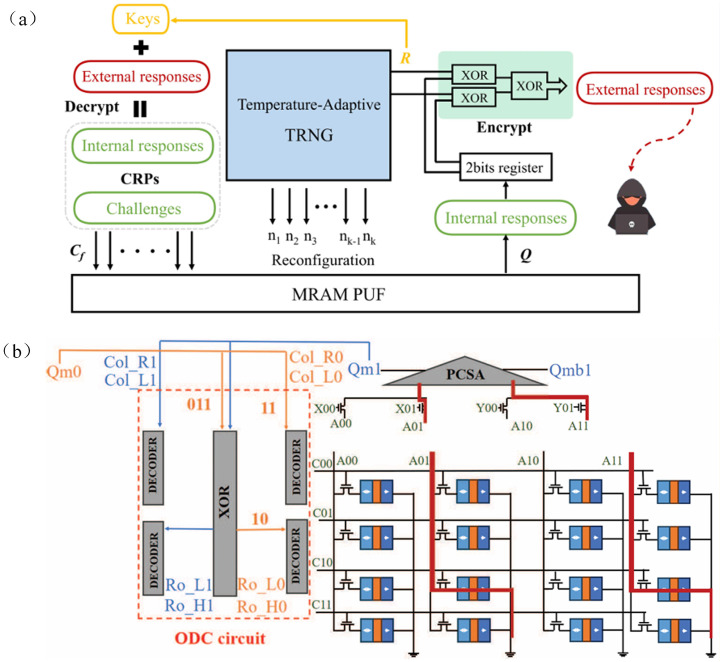
MRAM in security applications. (**a**) Temperature−adaptive TRNG-encrypted MRAM−PUF architecture, where a TRNG is tightly coupled with the MRAM-PUF to dynamically mask intrinsic responses through XOR−based encryption and reconfiguration signals, enhancing resistance against modeling and replay attacks. Reprinted from Ref. [134]. (**b**) Double−layer dynamic challenge cross−selection STT−MRAM PUF with an ODC, which increases the nonlinearity of the challenge−response relationship and improves robustness and security against machine−learning-based attacks. Reprinted from Ref. [135].

In the domain of random number generation, Suresh et al. developed a high-entropy STT-MRAM TRNG that exploits thermally induced variations in MTJ switching latency to achieve entropy rates approaching 1 bit per sample, while satisfying NIST SP800-22 and AIS-31 statistical test suites under process, voltage, and temperature variations [133]. Similarly, Dai et al. proposed a temperature-adaptive TRNG-encrypted MRAM-PUF architecture, in which intrinsic PUF responses are dynamically masked by TRNG-generated randomness to improve resistance against machine-learning-based modeling and replay attacks in thermally unstable environments [134]. In Figure 9a, the TRNG is tightly coupled with the MRAM-PUF, where the internally generated responses are encrypted through XOR-based operations before being exposed externally, while reconfiguration signals derived from the TRNG continuously perturb the challenge–response mapping. The design, validated in a 65 nm CMOS process using a compact 2T–1MTJ cell, demonstrates strong randomness and improved robustness while maintaining sub-5 μW power consumption suitable for ultra-low-power edge devices.

In parallel, MRAM-based PUF designs have been extensively explored to enhance hardware security. Adel et al. demonstrated an MRAM-PUF architecture with improved resistance to deep-learning-based attacks, where the prediction accuracy approaches random guessing for binary responses [136]. Wu et al. further proposed a double-layer dynamic challenge cross-selection STT-MRAM PUF in Figure 9b, where an obfuscation decode circuit (ODC) is inserted between two MRAM arrays to increase the nonlinearity of the challenge–response relationship, thereby improving robustness and security [135]. In addition, Lee et al. demonstrated spintronic PUFs based on field-free SOT switching, which provide stable responses under thermal stress and process variations [137]. These studies collectively demonstrate that MRAM-based PUFs can achieve both high entropy and strong resistance to modeling attacks.

MRAM’s inherent physical randomness, thermal fluctuation, and process-induced variability, once considered design challenges, are now actively leveraged for robust, lightweight, and tamper-resistant hardware security primitives, positioning MRAM as security-enabling memory beyond mere data storage.

## 4. Challenges

Despite being widely regarded as a promising cornerstone of next-generation memory technologies—owing to its intrinsic advantages such as non-volatility, high-speed operation, and theoretically high endurance—MRAM continues to confront a set of deeply rooted, multifaceted challenges that hinder its transition from niche markets to widespread commercial deployment. These challenges arise primarily from the unique interplay between MRAM’s physical switching mechanisms, complex material systems, and process integration constraints. Broadly, they can be categorized into four fundamental and interdependent domains that collectively define the current technological bottlenecks impeding large-scale MRAM commercialization.

### 4.1. The Inherent Trade-Offs in Write Performance

A fundamental limitation in MRAM technology lies in the conflicting requirements between write speed and energy efficiency. STT-MRAM, for example, requires a high current density to induce STT sufficient for deterministic magnetization reversal in nanosecond-scale timeframes. However, this directly results in excessive dynamic power consumption, which undermines MRAM’s energy advantage in low-power systems [138]. Conversely, reducing write current to save energy increases switching latency and worsens the WER, especially in scaled nodes where thermal stability becomes weaker [53]. This trade-off is rooted in the stochastic nature of spin-torque switching: a lower write current reduces the spin angular momentum delivered to the free layer, making the switching process more susceptible to thermal fluctuations and process-induced variations in anisotropy, resistance-area product, and critical current [139]. This speed–power paradox remains a core bottleneck in reconciling MRAM’s theoretical performance benefits with practical deployment in energy-constrained systems such as mobile devices, IoT nodes, and edge-computing platforms. Recent studies further confirm this inherent trade-off: experimental characterizations of perpendicular STT-MRAM report that lowering write current increases the probability of thermally activated failures and broadens the switching-time distribution [140], while device-level analyses show that scaled MTJs require even higher current densities to maintain acceptable energy barriers, exacerbating power penalties [141]. Moreover, write-reliability modeling indicates that sub-nanosecond switching under low-voltage bias dramatically elevates the WER tail, limiting applicability in energy-limited systems [53]. The dependence of WER on write voltage and MTJ size further indicates that aggressive scaling narrows the reliable write window of STT-MRAM [139]. Reference-layer stray fields can also distort the energy landscape of perpendicular MTJs and increase write-error probability, especially in scaled devices [142]. Therefore, write optimization cannot be treated only as a circuit-level problem; it requires co-optimization of the magnetic free layer, tunnel barrier, write pulse scheme, and error-tolerant architecture [140,141,142].

### 4.2. Process Compatibility and Integration Complexity

Although MRAM is often promoted as a CMOS-compatible memory, real-world implementation reveals non-trivial integration challenges, especially at advanced nodes. The fabrication of MTJs—the key storage elements in MRAM—requires specialized materials such as CoFeB and MgO and demands tight process control of ultra-thin layers (<2 nm) to ensure tunneling magnetoresistance consistency and thermal stability [141]. These steps are typically inserted during the BEOL stage, introducing contamination risks and yield loss when co-integrated with logic transistors [143]. In practice, MRAM integration is not merely the insertion of an additional memory layer; it requires precise control of magnetic multilayer deposition, interface roughness, sidewall damage during etching, post-etch cleaning, and annealing conditions [32]. Small deviations in MgO thickness, CoFeB crystallization, or reference-layer coupling can translate into large distributions in resistance, *TMR*, switching current, and retention [141]. Furthermore, the need for additional lithography masks, annealing steps, and magnetic shielding in SOT-MRAM further complicates process flows and increases fab cost [144]. For emerging FinFET or gate-all-around (GAA) platforms, MRAM integration is even more constrained due to limited thermal budgets and metal pitch congestion, limiting design flexibility in high-density SoCs. These challenges are particularly pronounced for SOT-MRAM, where the spin-source layer must simultaneously provide high charge-to-spin conversion efficiency, low resistivity, BEOL thermal stability, and compatibility with dense interconnect routing [145,146]. The 300 mm integration of SOT-MRAM has demonstrated the feasibility of CMOS-compatible fabrication, but it also highlights the need to control SOT material stacks, patterning damage, and array-level process uniformity [145]. Field-free perpendicular SOT-MRAM further requires additional symmetry-breaking designs, which increase integration complexity compared with conventional two-terminal STT-MRAM [146].

### 4.3. Cost, Yield, and Commercialization Challenges

Another critical issue is cost-effectiveness and manufacturing yield. While MRAM benefits from shrinking cell sizes in theory, in practice, the write current scaling is suboptimal, and the MTJ stack thickness cannot be reduced below physical thresholds (typically 1–1.5 nm) without sacrificing data retention [147]. These constraints hinder the adoption of aggressive scaling, limiting MRAM’s competitiveness against mature alternatives like embedded Flash, RRAM, or even DRAM in terms of bit cost per mm^2^ [148]. In addition, low write margins and process variation sensitivity often necessitate error-correcting codes or redundancy schemes, which further reduce usable yield and increase area overhead [149]. From a commercialization perspective, MRAM must compete not only on intrinsic device metrics, such as speed and endurance, but also on macro-level yield, peripheral-circuit overhead, test cost, and qualification reliability across temperature and lifetime conditions [32]. As a result, the unit cost of MRAM chips remains high, especially in high-density configurations, delaying market penetration beyond niche applications. This is one reason why current MRAM adoption is strongest in embedded non-volatile memory, automotive microcontrollers, industrial systems, and specialty memories, whereas direct cost-per-bit competition with high-density DRAM or NAND Flash remains difficult [32].

### 4.4. Physical and Architectural Limits to Density Scaling

MRAM scaling is ultimately limited by fundamental spintronic and magnetic material constraints. For instance, as cell sizes shrink, the thermal stability factor Δ (proportional to volume) decreases, resulting in increased susceptibility to spontaneous switching and retention loss, especially at high temperatures. In single-digit-nanometer MTJs, shape anisotropy has been revisited as a possible route to improve thermal stability, but it also introduces new constraints on material design, patterning accuracy, and switching current control [150]. This imposes a trade-off between switching energy, data retention, and device reliability, which becomes more severe in sub-20 nm nodes. Additionally, dipolar field interactions and write disturb effects become prominent in densely packed MTJ arrays, requiring complex shielding or isolation strategies [53]. Reference-layer stray fields and neighboring-cell magnetic interactions can further modify the switching probability and WER distribution in scaled perpendicular STT-MRAM arrays [142]. From an architectural perspective, multi-level cell designs are difficult to implement reliably in MRAM due to its binary switching nature, further limiting its density roadmap relative to NAND Flash or DRAM. Consequently, MRAM still falls short of delivering multi-gigabit density in cost-competitive formats.

## 5. Future Directions and Opportunities

The future development of MRAM is being driven by coordinated advances across materials, devices, architectures, and system-level integration. Several key research directions are emerging.

### 5.1. Exploration of New Material

Recent progress in MRAM has been strongly driven by advances in spintronic materials, particularly those enhancing spin–orbit torque efficiency and reducing switching energy. Emerging materials such as heavy metals and topological insulators with large spin Hall angles, orbital Hall materials, and engineered multilayer stacks enable lower write current and faster switching. Weyl semimetals and antiferromagnetic materials are also being investigated as alternative sources of spin–orbit torque because of their unconventional electronic structures, interfacial spin accumulation, or symmetry-dependent spin polarization [151]. For SOT-MRAM, the key material challenge is no longer only to maximize the spin Hall angle, but also to balance charge-to-spin conversion efficiency, resistivity, interface transparency, thermal stability, and compatibility with back-end-of-line processing [152]. In addition, two-dimensional magnetic materials and van der Waals heterostructures are being explored for ultra-scaled MRAM devices with improved tunability and energy efficiency [153,154]. Graphene, h-BN, transition-metal dichalcogenides, and van der Waals magnets may function as tunnel barriers, spin-filter layers, magnetic electrodes, or interface-engineering layers. Their atomically thin structures and dangling-bond-free interfaces provide opportunities for reducing interfacial disorder and electrically controlling spin transport and magnetism [155,156]. Orbitronic materials, which exploit orbital angular momentum rather than only spin angular momentum, provide another emerging route for generating angular-momentum currents in materials that may not possess strong spin–orbit coupling [157]. The generated orbital current can be converted into spin angular momentum in an adjacent magnetic layer and exert an orbital torque on the magnetization [158].

Despite these advances, most MRAM concepts based on two-dimensional and quantum materials remain at the material, single-device, or proof-of-concept stage. Wafer-scale synthesis, interface contamination, environmental and thermal stability, device-to-device reproducibility, nanoscale patterning, and CMOS back-end compatibility remain major barriers to large-scale memory integration [155,156]. These developments suggest that future improvements will increasingly rely on material–device co-optimization to overcome fundamental energy–speed trade-offs. Therefore, future MRAM material research is expected to move from simple material substitution toward interface-level and stack-level engineering, where magnetic anisotropy, damping, spin transparency, and thermal robustness are optimized simultaneously.

### 5.2. Novel Device Architectures

In parallel with material innovation, MRAM device architectures continue to evolve toward higher performance and scalability. Field-free SOT-MRAM, canted magnetization structures, and two-terminal SOT devices have demonstrated improved write efficiency and simplified integration [159,160]. Among these directions, field-free SOT switching is particularly important because practical SOT-MRAM arrays cannot rely on an external magnetic field for deterministic operation [161]. Meanwhile, perpendicular magnetic anisotropy (PMA) and composite free-layer designs further enhance thermal stability and scalability at advanced nodes [162]. Beyond conventional two-terminal STT-MRAM and three-terminal SOT-MRAM, future devices may also employ hybrid STT–SOT switching, voltage-assisted switching, or engineered free-layer structures to reduce write energy without sacrificing retention. These emerging device concepts provide a flexible foundation for extending MRAM into high-speed cache and embedded memory applications. However, their practical value will depend on whether device-level advantages can be preserved after array integration, where cell footprint, selector design, write disturbance, thermal budget, and peripheral-circuit overhead become equally important.

### 5.3. Integration with AI Hardware

MRAM is increasingly being integrated into AI hardware platforms, particularly in edge-AI processors and CIM architectures [163]. Recent silicon demonstrations of MRAM-based near-memory computing processors and heterogeneous AI systems have shown substantial improvements in energy efficiency and latency for real-time inference workloads [130]. These results indicate that MRAM is moving beyond conventional non-volatile storage and becoming an important component in energy-efficient AI hardware. Future MRAM-AI integration is expected to focus on edge and embedded inference, where low standby power, fast wake-up, and local weight storage are critical. Non-volatile memory technologies can reduce leakage energy and provide efficient on-chip storage for neural network parameters [164]. MRAM is also attractive for normally-off and always-on intelligent systems because model weights and system states can be retained without refresh power. Recent heterogeneous SoC studies further show that tightly coupled MRAM-based memory near neural engines can improve throughput and energy efficiency [130]. Another key direction is MRAM-based near-memory AI acceleration. A 22 nm nonvolatile AI-edge processor using a 47.25 Mb compressed-computing STT-MRAM macro achieved 21.4 TFLOPS/W, demonstrating the potential of large-capacity MRAM macros for energy-efficient edge inference [163]. In addition, binary, ternary, and low-bit-width neural networks are more compatible with MRAM-based accelerators because they can better tolerate device variation and limited resistance precision [165]. Future research should therefore emphasize device–circuit–architecture co-optimization, variation-aware mapping, low-power sensing, flexible precision support, and heterogeneous memory hierarchy design [164]. MRAM is more likely to complement existing GPU- and SRAM-based AI hardware as a non-volatile, low-standby-power, and instant-on memory component rather than directly replacing them.

### 5.4. In-Memory Computing

In-memory computing provides a further direction for MRAM-based hardware acceleration. More recently, MRAM crossbar arrays have been experimentally demonstrated for in-memory computing, showing that MRAM can support analog multiply–accumulate operations by exploiting resistance-state summation rather than acting only as a passive non-volatile storage element [166]. Unlike near-memory computing, where computation is performed by logic located adjacent to the memory array, MRAM-based in-memory computing executes selected arithmetic or logic operations directly within the array, further reducing the movement of weights, operands, and intermediate data [167]. These results indicate that MRAM is transitioning from a passive storage element to an active computing medium, where device characteristics, circuit design, and algorithm robustness must be jointly optimized. Analog MRAM-based IMC provides high parallelism and energy efficiency but remains sensitive to resistance variation, read noise, low device resistance, and analog-to-digital conversion overhead [167]. Digital MRAM-based IMC offers higher numerical precision and robustness, although additional sensing, digitization, accumulation, and control circuits increase circuit complexity and hardware overhead [168].

Therefore, future MRAM-based IMC will require device–circuit–algorithm co-design, improved read margin, reduced write energy, variation-aware training, flexible numerical precision, and scalable integration with CMOS peripheral circuits.

### 5.5. Hybrid Architectures

Due to current density and scalability constraints, MRAM is increasingly considered in hybrid memory and computing systems. In such systems, MRAM serves as a non-volatile buffer or fast log layer, while DRAM or Flash provides bulk storage capacity. This organization improves the balance among data retention, boot-up latency, and endurance in SoCs, especially for edge-computing applications. At the memory-system level, hybrid architectures are attractive because they allow MRAM to be placed where its non-volatility, low standby power, and high endurance provide the greatest system-level benefit, rather than forcing it to compete directly with DRAM or NAND Flash on raw bit density. Hybrid cache and memory hierarchies can combine the low write latency of SRAM, the density advantage of STT-MRAM, and the capacity advantage of DRAM or Flash, thereby reducing leakage power while avoiding the write-energy penalty of using MRAM for all memory levels [169]. At the architecture level, RRAM/MRAM hybrid computing-in-memory architecture integrates heterogeneous memory media through cross-array data buffering and bitwise fusion mechanisms. A dynamic system control module enables adaptive switching between high-energy-efficiency and high-accuracy computing modes, thereby alleviating the performance bottlenecks of single-memory-medium designs. Hybrid architectures also introduce new design problems, including data placement, wear balancing, memory consistency, compiler support, and runtime scheduling. Future work should therefore treat MRAM not only as a device replacement, but as part of a heterogeneous memory hierarchy co-designed with software and workload characteristics.

### 5.6. Commercialization Landscape and Ecosystem Growth

The commercialization of MRAM has progressed rapidly, with embedded STT-MRAM already deployed in automotive and IoT microcontrollers. Foundries have demonstrated mature eMRAM technologies at advanced nodes, while ongoing development of SOT-MRAM aims to achieve SRAM-class performance for high-speed application. At the same time, the MRAM ecosystem—including design tools, IP support, and manufacturing infrastructure—is expanding, facilitating broader adoption in both edge and high-performance computing systems. This ecosystem-level progress is important because MRAM commercialization depends not only on MTJ performance, but also on qualified process design kits, reliable memory compilers, built-in self-test schemes, error-correction support, and long-term reliability qualification. In the near term, the most realistic commercial path for MRAM is likely to be application-specific deployment in embedded non-volatile memory, industrial and automotive microcontrollers, radiation-tolerant systems, and low-power edge devices. In the longer term, if SOT-MRAM and related material platforms can reduce write current while maintaining manufacturability, MRAM may expand toward high-speed cache, compute-near-memory, and normally-off computing platforms.

## 6. Conclusions

MRAM exhibits strong potential for future memory and computing systems. Its non-volatility, high endurance, nanosecond-scale write speed, and CMOS compatibility make it a strong candidate for embedded memories, edge devices, high-reliability electronics, intelligent computing hardware, and hardware security. This review summarized MRAM from the perspectives of device fundamentals, technology evolution, applications, challenges, and future opportunities. MRAM stores information through magnetic states in MTJs, which enables non-volatility and high endurance but also introduces challenges in magnetic stability, switching current, tunnel-barrier reliability, and process integration. The evolution from Toggle-MRAM to STT-MRAM and SOT-MRAM shows the continuous effort to improve write efficiency, scalability, and reliability. Toggle-MRAM proved the feasibility of magnetic non-volatile storage, STT-MRAM enabled compact 1T–1MTJ cells and commercial embedded memory, and SOT-MRAM further improved speed and endurance through separated read/write paths.

MRAM is promising for embedded SoCs, edge devices, high-reliability electronics, AI hardware, and near-memory or in-memory computing. It can reduce standby power, support fast state retention, and improve system-level energy efficiency. However, broader adoption still requires solutions to write current reduction, WER control, retention scaling, CMOS back-end integration, cost, yield, and peripheral-circuit overhead. Future MRAM development will depend on co-optimization across materials, MTJ stacks, device architectures, circuits, processes, and application-specific systems. In the near term, embedded non-volatile memory, automotive electronics, industrial systems, and edge devices remain the most realistic commercial directions. In the longer term, SOT-MRAM, voltage-assisted switching, new spin–orbit materials, and MRAM-based computing may expand MRAM into a broader spintronic hardware platform.

## Figures and Tables

**Table 1:** Timeline of MRAM production.

Year	Manufacturer	Write Mechanism	Process	Capacity	Speed	Voltage/Current/Energy
2005	Sony [53]	STT-MRAM	180 nm	4 kbit	W ≈ 2 ns	W ≈ 200 μA
2006	Freescale [54]	Toggle-MRAM	180 nm	4 Mbit	W ≈ 35 ns	I ≈ 5–10 mA
2008	MagIC [55]	STT-MRAM	130 nm	64 kbit	W ≈ 20 ns	I ≈ 1 mA
2009	NEC [56]	STT-MRAM	90 nm	32 Mbit	W/R ≈ 12 ns	R ≈ 57 mW; W ≈ 70 mW
2010	Tohoku Univ./Fujitsu [57]	STT-MRAM	130 nm	16 kbit	R ≈ 9 ns; W ≈ 9–10 ns	I ≈ 0.4–0.87 mA
2010	Toshiba [58]	STT-MRAM	65 nm	64 Mbit	R/W ≈ 30 ns	I ≈ 10 μA
2010	Grandis [59]	STT-MRAM	54 nm	64 Mbit	R ≈ 20 ns	I ≈ 140 μA
2010	Hitachi [60]	STT-MRAM	150 nm	32 Mbit	R ≈ 32 ns; W ≈ 40 ns	I ≈ 300 μA
2010	IBM [61]	STT-MRAM	—	4 kbit	W ≈ 50 ns	—
2011	Qualcomm [62]	STT-MRAM	45 nm	1 Mbit	R ≈ 8 ns	VDD ≈ 1.8 V
2012	Everspin [63]	STT-MRAM	90 nm	64 Mbit	W ≈ 50 ns	VDD ≈ 1.5 V
2013	TSMC [64]	STT-MRAM	40 nm	1 Mbit	R ≈ 10 ns	I ≈ 281–283 μA
2013	NEC/Tohoku Univ. [65]	STT-MRAM	90 nm	1 Mbit	R ≈ 1.5 ns; W ≈ 2.1 ns	VDD ≈ 1.3 V
2013	IBM/TDK [66]	STT-MRAM	90 nm	8 Mbit	W ≈ 1.5 ns	—
2013	Toshiba [67]	STT-MRAM	65 nm	512 kbit	W/R ≈ 8 ns	R ≈ 4 mW; W ≈ 15 mW
2013	Toshiba [68]	STT-MRAM	65 nm	1 Mbit	W/R ≈ 4 ns	R ≈ 17.8 mW; W ≈ 46.5 mW
2014	TDK [69]	STT-MRAM	90 nm	8 Mbit	R ≈ 4 ns; W ≈ 4.5 ns	—
2015	IBM/TDK [70]	STT-MRAM	90 nm	8 Mbit	R/W < 70 ns	R < 100 mV; W ≈ 600 mV
2015	Toshiba [71]	STT-MRAM	65 nm	1 Mbit	R ≈ 3.3 ns; W ≈ 3 ns	R ≈ 71.2 μJ/MHz; W ≈ 166.2 μJ/MHz
2016	Toshiba/Tohoku Univ. [72]	STT-MRAM	65 nm	4 Mbit	R ≈ 3.3 ns	VDD ≈ 1.25 V
2016	Everspin/GF [33]	STT-MRAM	40 nm	256 Mbit	W ≈ 50 ns	—
2017	Samsung [73]	STT-MRAM	28 nm	8 Mbit	R ≈ 10 ns	—
2018	TSMC [74]	STT-MRAM	28 nm	1 Mbit	R ≈ 2.8 ns; W ≈ 20 ns	VDD ≈ 1.2 V
2019	Everspin [75]	STT-MRAM	28 nm	1 Gbit	—	—
2019	Samsung [34]	STT-MRAM	28 nm	1 Gbit	—	—
2019	Intel [76]	STT-MRAM	55 nm	16 Mbit	R ≈ 4 ns; W ≈ 20 ns	—
2020	IBM [77]	STT-MRAM	40 nm	32 Mbit	W ≈ 3 ns	—
2020	Samsung/ARM [78]	STT-MRAM	28 nm	128 Mbit	R ≈ 33 ns	R ≈ 1.2 pJ/bit
2022	Samsung [35]	STT-MRAM	14 nm	16 Mbit	—	W ≈ 25 pJ/bit
2022	Everspin [36]	STT-MRAM	—	8–64 Mbit	—	—
2023	Beihang Univ. [51]	SOT-MRAM	—	1 Kbit	W ≈ 20 ns	WER down to $10^{-6}$
2024	Renesas [79]	STT-MRAM	22 nm	10.8 Mbit	>200 MHz random-read frequency (~4.2 ns class)	Write throughput 10.4 MB/s
2025	NYCU/TSMC/ITRI [52]	SOT-MRAM	—	64 Kbit	Switching 1 ns	TMR 146%; retention > 10 years

**Table 2:** Comparison of key characteristics of conventional and emerging memory technologies.

Feature/ Metric	SOT-MRAM	STT-MRAM	Toggle-MRAM	3D NAND Flash	eFlash	DRAM	SRAM
Write Mechanism	Spin–orbit torque (current-induced spin Hall effect)	Spin-transfer torque (current-induced magnetization switching)	Magnetic-field-induced toggle switching	Charge storage in charge-trap	Hot-electron injection/tunneling	Charge storage in capacitor	Bistable latch circuit
Write Speed	0.3–2 ns	5–50 ns	20–100 ns	100 µs–1 ms	1–10 µs	10–50 ns	<1 ns
Read Speed	<5 ns	5–20 ns	20–50 ns	50–100 µs	50–200 ns	10–30 ns	<1 ns
Write Power Consumption	Very low (sub-pJ/bit)	Low (pJ/bit)	High (nJ/bit)	High	Moderate	Moderate	High
Endurance (Write Cycles)	>10^15^	10^10^–10^15^	>10^15^	10^13^–10^15^	10^4^–10^6^	-	-
Integration Density	High	Moderate–High	Low	Very High	Moderate	High	Low
Non-volatility	√	√	√	√	√	×	×
CMOS Compatibility	Excellent	Excellent	Good	Limited	Limited	Excellent	Excellent
Operating Voltage	0.6–1.2 V	0.8–1.2 V	1.5–3.3 V	3–5 V	1.8–3.3 V	1.0–1.2 V	1.0–1.2 V
Reference	[19,33,79]	[19,33,79]	[1,33,53,79,80]	[81]	[82]	[83,84]	[83,84]

## Data Availability

No new data were created or analyzed in this study. Data sharing is not applicable to this article.
